# Study on the Impact of Diluent Dosages on the Epoxy–Polythiol Self-Healing System

**DOI:** 10.3390/polym17040538

**Published:** 2025-02-19

**Authors:** Jiajia Sheng, Yang Guo, Xin Pang, Wenjing Ma, Hailu Yang, Yalin Liu, Linbing Wang, Shanglin Song

**Affiliations:** 1 National Center for Materials Service Safety, University of Science and Technology Beijing, Beijing 102206, China; shengjj@xs.ustb.edu.cn (J.S.); guoyang@ustb.edu.cn (Y.G.); yalinau@ustb.edu.cn (Y.L.); 2 Research Institute of Urbanization and Urban Safety, School of Civil and Resource Engineering, University of Science and Technology Beijing, Beijing 100083, China; d202210025@xs.ustb.edu.cn; 3 State Grid Qinghai Province Power Company Xining Power Supply Company, Xining 810003, China; 18697157363@163.com; 4 School of Environmental, Civil, Agricultural and Mechanical Engineering, University of Georgia, Athens, GA 30602, USA; 5 Scientific Observation and Research Base of Transport Industry of Long Term Performance of Highway Infrastructure in Northwest Cold and Arid Regions, Gansu Provincial Highway Development Group Co., Ltd., Lanzhou 730030, China; 17801052381@163.com

**Keywords:** diluent, microcapsules, self-healing system, thermodynamics, Young’s modulus

## Abstract

Self-healing technology is an effective method for enhancing the crack resistance of cement-based composites. This study focuses on the impact of the environmentally friendly diluent C12-14 alkyl glycidyl ether (AGE) on the performance of the epoxy resin–polythiol (rimethylolpropane tris (3-mercaptopropionate), TMPMP) self-healing system, examining core fluidity, microcapsule properties, molecular dynamics, and the mechanical properties of cured products. The results show that as the AGE dosage increases, the particle size distribution of microcapsules becomes more concentrated, and the dispersion of particles is improved. Fourier-transform infrared spectroscopy confirms the successful encapsulation of E-51 and AGE. Microcapsules maintain structural integrity at high temperatures of 423.15 K. The onset thermal degradation temperature of the mixture shows an increasing trend with reduced AGE dosage. Specifically, TMPMP_35%_ exhibits an onset degradation temperature of 370.95 K, while that of TMPMP_20%_ is increased by 57.57% compared to TMPMP_35%_. Conversely, the initial and peak temperatures of the curing reaction decrease with less AGE incorporation. Thermodynamic analysis reveals that activation energy (E) initially increases and then decreases with increasing AGE. The frequency factor (A) correlates positively with the heating rate, indicating that the curing reaction’s reactivity is closely linked to heating rate. Minor variations in the reaction rate constant (k) indicate that the self-healing system maintains stable reactive activity at low temperatures. Notably, the AGE dosage significantly affects the rigidity of the self-healing system; the average Young’s modulus inversely correlates with AGE dosage, with the most substantial decrease of 5.88% occurring when AGE increases from 30% to 35%. This study offers insights into optimizing diluent ratios to balance self-healing and mechanical properties, essential for developing high-performance self-healing cement materials.

## 1. Introduction

Cement-based materials are among the most critical building materials in contemporary infrastructure, known for their high mechanical performance and good durability [1,2]. However, a significant vulnerability of cement-based materials is their propensity to crack [3]. Using reinforced concrete as an example, microcracks develop in the structure under applied loads and can propagate into larger cracks under continued loading. Although these cracks intersect with the reinforcement bars, causing separation between the steel and concrete at the crack sites and thereby inducing tensile stresses in the steel [4], the presence of microcracks may facilitate the ingress of unwanted pollutants, salts, and chemicals that corrode the structure. This, in turn, significantly reduces the durability and service life of the structure [5]. Furthermore, the formation of microcracks occurs during the early stages of construction and persists throughout the entire lifecycle of the structure. Preventing the development of cracks is a crucial task for ensuring structural durability and managing associated aesthetic consequences. However, detecting and intervening in the progression of cracks from micro-cracks to macro-cracks manually is challenging. Therefore, employing the concept of self-healing to address this issue can mitigate the expansion of micro-cracks into macro-cracks in cementitious materials [6,7].

The self-healing of cement-based materials is primarily divided into two categories: autogenous healing and autonomous healing [8]. Autogenous healing is a phenomenon based on the chemical reactions between water penetration and unhydrated cement particles [9]. However, the hydration of cement particles requires the presence of water and is a prolonged self-healing process [10,11]. Consequently, researchers have introduced the concept of autonomous healing. This technique involves adding specially designed additives to cementitious materials, enabling them to self-heal autonomously, akin to living organisms. Microcapsule encapsulation of healing agents is one of the most common self-healing technologies in cement-based materials [12,13,14,15,16]. Researchers utilize various encapsulation techniques to encapsulate the core material within a shell [6,17,18,19,20], employing triggers such as pH [20], light [21], electromagnetic fields [16], microwaves [22], ultrasound [23], and mechanical activation [24] to control the release of the healing agent. After microcapsules rupture, capillary action facilitates the flow of core materials into microcracks [25]. This provides timely repair of micro-cracks that occur within the structure, ensuring its integrity.

Researchers typically focuses on the effects of the fabrication principles and controlled release mechanisms on the intrinsic properties of microcapsules [23,26,27,28,29], as well as the analysis of the repair effects on cement slurries containing microcapsules [30,31,32,33,34]. In particular, research on the emulsifiers and diluents used during the preparation of epoxy resin microcapsules has primarily concentrated on exploring emulsifiers [35,36,37]. Diluents are generally considered to merely reduce the viscosity of adhesives, enabling effective penetration and filling of cracks [38,39,40]. In fact, diluents not only serve to dilute the core material but also influence the preparation of epoxy resin microcapsules, as well as the curing molecular dynamics and mechanical properties of the entire epoxy resin hardener system [19,41]. However, research on the impact of diluent dosage on the preparation of epoxy resin microcapsules and the epoxy resin hardener system remains relatively limited.

Currently, numerous healing agents are suitable for use in cement-based materials [20,42,43,44,45,46], and the design and selection of these agents must meet specific criteria. They should be environmentally friendly; possess low viscosity, high fluidity, and low shrinkage; and exhibit certain chemical resistance and the ability to cure rapidly at or below ambient temperatures [47]. Epoxy resin E-51, as a non-toxic and cost-effective adhesive, meets the conditions of low viscosity and high fluidity under the influence of diluents. It should be allowed to cure within several hours to days at room temperature. In the presence of a catalyst at temperatures between 130 °C and 180 °C, it can heal cracks within 1.5 h with a healing efficiency exceeding 80% [48,49,50,51]. When used with a thiol curing agent, the resulting self-healing system can cure rapidly at −10 °C [52,53], and the cured products display excellent mechanical strength, environmental stability, and low shrinkage [54]. Therefore, epoxy resin E-51 becomes an ideal choice for the core material of microcapsules [55,56,57]. Butyl glycidyl ether (BGE) [58,59] and C12-14 alkyl glycidyl ether (AGE) [36,41] have been demonstrated to be the best diluents for epoxy resins, with both AGE and BGE existing as liquids at room temperature. Compared to BGE, AGE exhibits significantly lower viscosity and a lower epoxy equivalent weight (approximately 0.3–0.4), with a mild and safe chemical form. Additionally, AGE shows low sensitivity to light, air, and humidity; possesses low toxicity; and can be safely stored at room temperature due to its high flash point.

Therefore, this study selected an environmentally friendly diluent suitable for large-scale industrial application—C12-14 alkyl glycidyl ether (AGE). Using core material research, microcapsule preparation, and performance evaluation of the curing system, this study assesses the impact of diluent addition on the preparation of epoxy resin microcapsules and the performance of the epoxy resin–polythiol self-healing system. The amounts of AGE added were 20%, 25%, 30%, and 35% by weight of the epoxy resin, with the epoxy resin (E-51) and polythiol (TMPMP) self-healing system mixed in a 1:1 ratio. The optimal ratio of diluent to epoxy resin was determined through microcapsule performance studies, thermodynamic analysis of the curing system, and mechanical property evaluations. This provides a reference for balancing self-healing capabilities and mechanical properties, thereby promoting the widespread application of microcapsule self-healing technology in engineering applications.

## 2. Materials and Methods

### 2.1. Materials

The epoxy resin (E-51) used as a healing agent, the defoamer n-octanol (AR, 99%), and the curing catalyst 2,4,6-tris (dimethylaminomethyl) phenol (DMP-30, AR, 95%) were supplied by the MACKLIN brand. C12-14 alkyl glycidyl ether (AGE, AR, epoxy value: 0.3–0.34) used as the diluent for the healing agent, the shell materials urea (AR, 99%) and formaldehyde (AR, 37% in H_2_O), the surfactant sodium dodecyl benzene sulfonate (SDBS, AR, 90%), the cleaning agent ethanol (AR, 75% in H_2_O), ammonium chloride (AR, 99.5%), resorcinol (AR, 99%), citric acid (AR, 99%), and the polythiol trimethylolpropane tris(3-mercaptopropionate) (TMPMP, AR, 85%) used as a curing agent were all provided by the MREDA brand. All the aforementioned materials were purchased from Beijing Mreda Technology Co., Ltd., located in Beijing, China.

### 2.2. Determination of Diluent Dosage of AGE

To study the effect of diluent addition on the viscosity of epoxy resin, the viscosity of the mixture was measured using a rotational viscometer (NDJ-9T) at room temperature (25 °C). Briefly, 30 g of E-51 and a specific amount of AGE were added to a beaker, thoroughly stirred, and then left to stand for 1–2 min until the liquid surface stabilized. Subsequently, the rotor of the rotational viscometer was placed into the beaker. The viscosity test for each additive ratio lasted 10 min, and the viscosity recorded by the viscometer after 10 min was taken as the final result.

**Figure 1:**
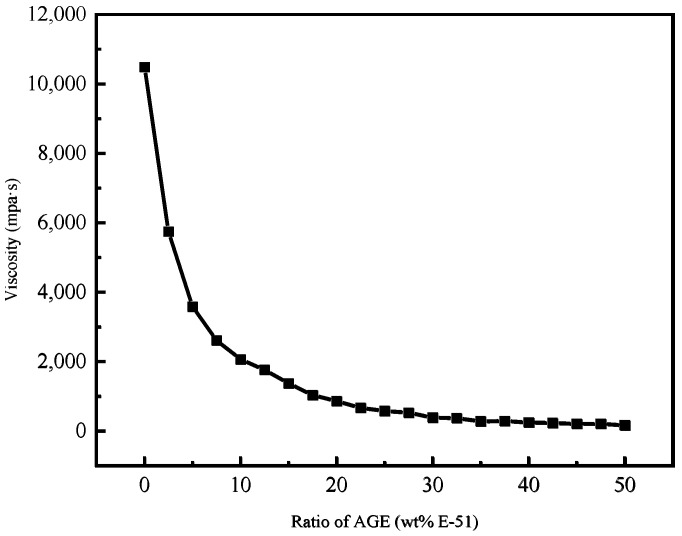
Effect of C12-14 alkyl glycidyl ether (AGE) on the viscosity of E-51.

As shown in Figure 1, as the proportion of AGE increased from 0% to 20%, the viscosity of the AGE/E-51 mixture significantly decreased. As the proportion of AGE further increased from 20% to 35%, the rate of viscosity reduction gradually slowed down. When the proportion of AGE exceeded 35%, the change in viscosity of the AGE/E-51 mixture was no longer significant, a phenomenon attributable to the good compatibility of epoxy groups and ether bonds in E-51. The addition of AGE weakened the intermolecular forces within E-51, reducing the overall stiffness of the mixture and thereby lowering its viscosity [60]. However, if the AGE dosage exceeds a specific level, its diluting effect no longer changes significantly. Therefore, based on the viscosity measurements of AGE, the optimal proportion of AGE to be mixed into E-51 should be between 20% and 35%. The dosages of AGE are added to the epoxy resin at ratios of 20%, 25%, 30%, and 35% of the epoxy resin mass, forming four different mixtures.

### 2.3. Preparation of Sample

**Figure 2:**
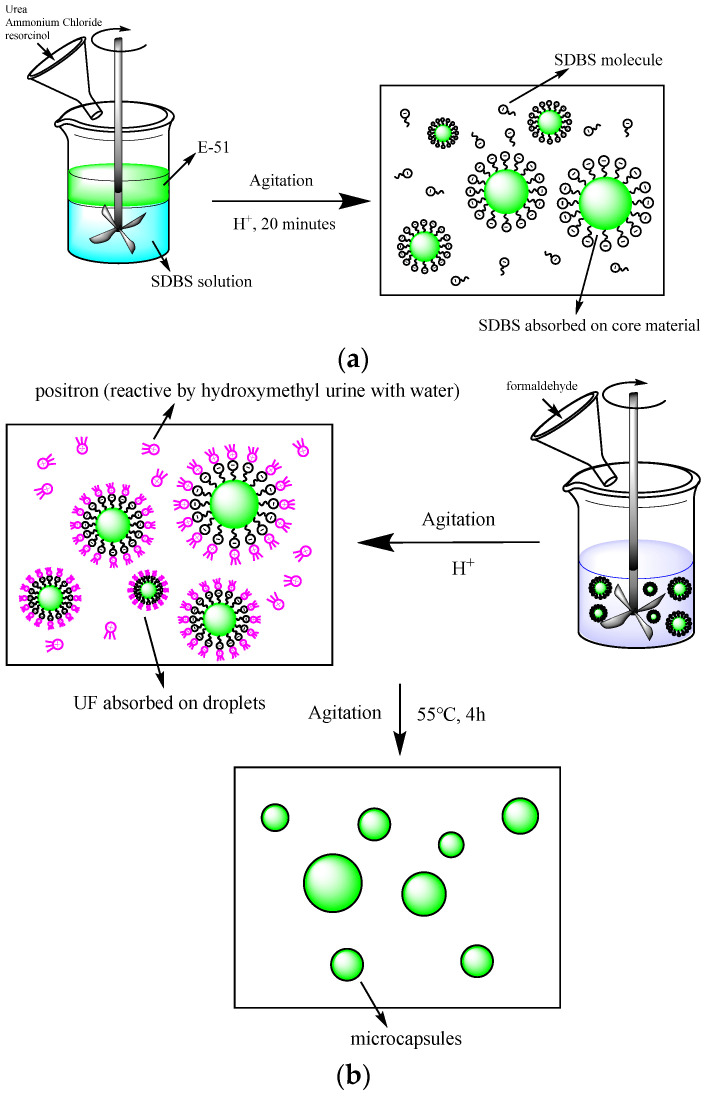
The synthesis process of self-healing microcapsules. (**a**) Emulsion of E-51; (**b**) Synthesis of microcapsules.

**Figure 3:**
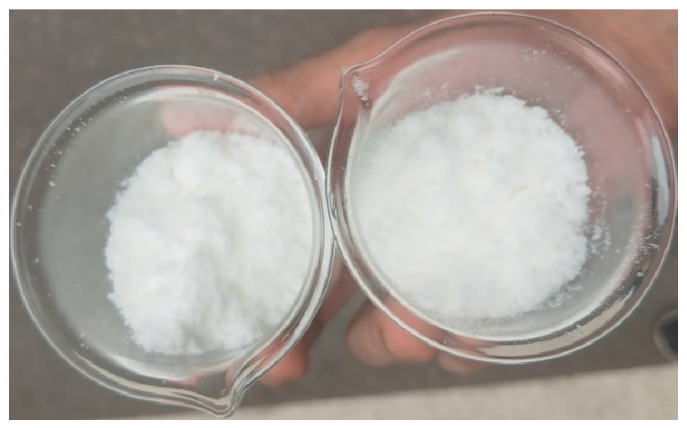
The sample of microcapsules with a diluent dosage of 30%.

In this study, AGE was incorporated into the epoxy resin at ratios of 20%, 25%, 30%, and 35% by weight of the epoxy resin, resulting in four different mixtures, designated as AGE_20%_, AGE_25%_, AGE_30%_, and AGE_35%_. These mixtures served as the core materials for microcapsules. Urea–formaldehyde resin was then synthesized through the chemical reaction between urea and formaldehyde to form the shell material of the microcapsules. The microcapsules, referred to as MCs_20%_, MCs_25%_, MCs_30%_, and MCs_35%_, were prepared by encapsulating the mixture core with the urea–formaldehyde resin shell using a one-step method [61]. The preparation process is illustrated in Figure 2. In a beaker, 250 mL of deionized water, 150 mL of 0.75 wt% SDBS solution, 10 g of urea, 1 g of ammonium chloride, and 1 g of resorcinol were added. Subsequently, a specific amount of epoxy resin and AGE mixture solution was incorporated. The stirring speed was set to 650 r/min, and the mixture was stirred for 20 min. The pH of the solution was adjusted to approximately 3.5. Once the emulsion stabilized, a 37% formaldehyde aqueous solution was added, and the temperature was raised to 55 °C, maintaining the reaction for 4 h. Figure 3 illustrates the epoxy resin microcapsules prepared with a diluent dosage of 30%, with the specific components listed in Table 1.

**Figure 4:**
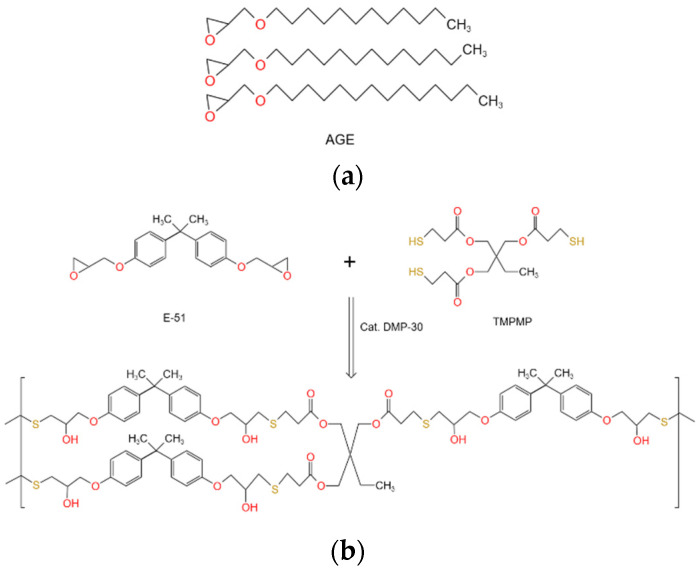
The structural formula and the curing reaction. (**a**) The structural formula of AGE; (**b**) Curing of E-51 and polythiol.

The epoxy–polythiol hybrid self-healing system was then created by thoroughly mixing each of these four mixtures with polythiol (equal in mass to the epoxy resin) and a curing catalyst (1/15 of the mass of the epoxy resin), resulting in mixtures labeled TMPMP_20%_, TMPMP_25%_, TMPMP_30%_, and TMPMP_35%_. It is important to note that when the molar ratio of thiol to epoxy is 1:1, a complete reaction can be achieved. Figure 4 shows the structural formulas of AGE, E-51, and TMPMP, as well as the curing reaction of epoxy–polythiol [47,62,63]. The molecular weights of E-51 and TMPMP are similar; thus, a 1:1 mass ratio is adopted for simplicity [64]. The molecular formula of E-51 contains two epoxy groups, while TMPMP has three thiol groups, ensuring a sufficient thiol dosage. Additionally, the dosage of epoxy groups in AGE is relatively low; thus, the likelihood of incomplete reactions due to insufficient curing agents is minimal.

### 2.4. Measurement Characterization

#### 2.4.1. Morphology and Size Characteristics of Microcapsules

To further investigate the impact of different ratios of AGE/E-51 on the surface morphology of the microcapsules, this study conducted surface morphology analysis using a scanning electron microscope (SEM, ZEISS EVO18, Carl Zeiss AG, Jena, Germany) on four different microcapsules: MCs_20%_, MCs_25%_, MCs_30%_, and MCs_35%_.

Additionally, the average diameter and particle size distribution of the microcapsules MCs_20%_, MCs_25%_, MCs_30%_, and MCs_35%_ were measured using a laser particle size analyzer (LPA, MASTERSIZER 3000, Malvern Panalytical, Malvern, UK). The procedure involved dispersing the microcapsule samples in deionized water, followed by precise measurement of the microcapsule particle diameters and distribution using laser scanning technology. The device automatically measures three times and then provides the average value of the three tests to evaluate the impact of the AGE addition on the size distribution of the microcapsules.

#### 2.4.2. Chemical Structure and Thermal Properties of Microcapsules

In this study, Fourier Transform Infrared Spectroscopy (FTIR-Nicolet IS50, Thermo Nicolet Corporation, Waltham, MA, USA) was used to perform spectral analysis on four types of microcapsules: MCs_20%_, MCs_25%_, MCs_30%_, and MCs_35%_. The FT-IR spectra of the samples in the absorption mode were recorded by scanning 32 times in the range of 400–4000 cm^−1^, thereby facilitating the identification of the chemical structures of each group microcapsule sample. About 1 mg of sample and about 100 mg of potassium bromide (KBr) were fully ground (the mass ratio of sample to KBr was about 1:100) and then imported into the mold. The sample was pressed into transparent sheets at 20 MPa with a press and then put into the instrument for testing. KBr is transparent to infrared light, which isolates the interaction between the sample and the light source during the experiment and provides a certain degree of dilution to ensure the accuracy of the spectra.

For the assessment of the thermal stability of the microcapsules, the thermal stability analysis was performed using a thermogravimetric analyzer (TGA, TA NETZSCH, Netzsch, Selb, Germany). Under protection of nitrogen flow, the samples were heated from 303.15 K to 773.15 K at a heating rate of 10 K/min.

#### 2.4.3. Thermal Properties of the Curing Products of Epoxy–Polythiol Self-Healing Systems

The thermal stability of the uncured TMPMP_20%_, TMPMP_25%_, TMPMP_30%_, and TMPMP_35%_ solutions was assessed using a thermogravimetric analyzer (TGA). Under the protection of nitrogen flow, the samples were heated from 303.15 K to 773.15 K at a heating rate of 10 K/min.

Differential scanning calorimetry (DSC2, Mettler Toledo, Switzerland) was used to study the effect of different AGE dosages and different heating rates on the kinetics of the curing reaction (characterizing the reactivity of the core materials). Tests were conducted within the temperature range from 293.15 K to 573.15 K under a nitrogen flow. The heating rates of 5 K/min, 10 K/min, 15 K/min, and 20 K/min were applied in accordance with standard testing procedures [65,66,67].

The curing process of the epoxy–polythiol self-healing system follows the Arrhenius equation [68], which is expressed as follows:

(1) $$\frac{\mathrm{da}}{\mathrm{dt}}{=\mathrm{Ae}}^{-E/\mathrm{RT}}{\left(1\;-\;a\right)}^{n}$$

where $\frac{\mathrm{da}}{\mathrm{dt}}$ is the curing rate; *a* is the degree of cure (%); *t* is the curing time (min); *A* is the pre-exponential factor (1/s); *E* is the activation energy of the curing reaction (J/mol); *R* is the universal gas constant (8.31 J/K·mol); *T* is the absolute temperature of the curing reaction (K); and *n* is the order constant of the curing reaction.

To further explore the nature of the curing reaction, the Kissinger and Crane equations were used to calculate related thermodynamic parameters. The Kissinger equation [69] is expressed as follows:

(2) $$\frac{d(\ln\frac{\beta }{T_{p}^{2}})}{d(\frac{1}{T})}=-\frac{E}{R}$$

where *T_p_* is the peak temperature, and *β* is the heating rate. When $\frac{E}{\mathrm{nR}}\gg {2T}_{p}$, considering the Crane equation [70], it is expressed as follows:

(3) $$\frac{d(\ln\beta )}{d(\frac{1}{T_{p}})}=-\frac{E}{\mathrm{nR}}$$

From the Kissinger and Arrhenius equations, the frequency factor *A* and the rate constant *k* can be determined.

(4) $$A=\frac{{\beta \mathrm{Ee}}^{{E/\mathrm{RT}}_{p}}}{{\mathrm{RT}}_{p}^{2}}$$

(5) $${k=\mathrm{Ae}}^{{-E/\mathrm{RT}}_{p}}$$

#### 2.4.4. Young’s Modulus of the Curing Products of Epoxy–Polythiol

To evaluate the Young’s modulus of the cured samples TMPMP_20%_, TMPMP_25%_, TMPMP_30%_, and TMPMP_35%_, drops of each uncured sample were placed on a glass slide on a tabletop. The slides were not touched during the curing process to ensure a smooth surface. After curing, the Young’s modulus of the cured products was measured using an atomic force microscope (AFM, Asylum Research MFP-3D, Oxford Instruments, Oxford, UK). A silicon probe with a resonance frequency of 70 Hz and a spring constant of 2 N/m was used in tapping mode, operating in repulsive mode to scan a 20 μm × 20 μm area of the cured samples.

The Young’s modulus is calculated using the Derjaguin–Muller–Toporov (DMT) model [71], which considers the adhesive force between the tip of the probe and the sample surface. This adhesion force is assumed to primarily act at the edges of the contact area. In this model, the interaction force *F* between the probe tip and the sample and the contact depth *δ* are measured using AFM experiments. From these measurements, the Young’s modulus of the sample is determined, which is expressed as follows:

(6) $${F=F}_{\mathrm{elastic}}+F_{\mathrm{adhesion}}$$

where *F* represents the interaction force between the probe tip and the sample (N). *F_elastic_* is the elastic force, and *F_adhesion_* is the adhesive force during the contact between the probe and the sample.

(7) $$F_{\mathrm{elastic}}=\frac{4}{3}E^{*}\sqrt{R}\delta^{3/2}$$

Here, *δ* represents the contact depth between the probe tip and the sample (m); *R* is the radius of curvature of the tip (m); and E* is the effective Young’s modulus between the tip and the sample (N/m^2^).

(8) $$\frac{1}{E^{*}}=\frac{{1-\nu }_{1}^{2}}{E_{1}}+\frac{{1-\nu }_{2}^{2}}{E_{2}}$$

Here, *E*_1_ and *E*_2_ are the Young’s modulus of the probe tip and the sample, respectively, while *v*_1_ and *v*_2_ are the Poisson’s ratios of the probe tip and the sample, respectively.

## 3. Results and Analysis

### 3.1. Influence of Different Diluent Concentrations of Microcapsules

#### 3.1.1. The Morphology and Size Distribution of Microcapsules

**Figure 5:**
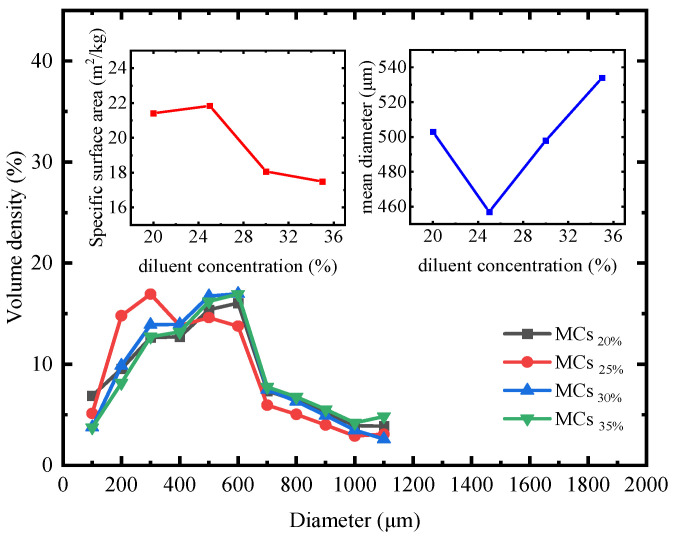
The effect of different AGE dosages on the particle size and specific surface area of microcapsules.

**Figure 6:**
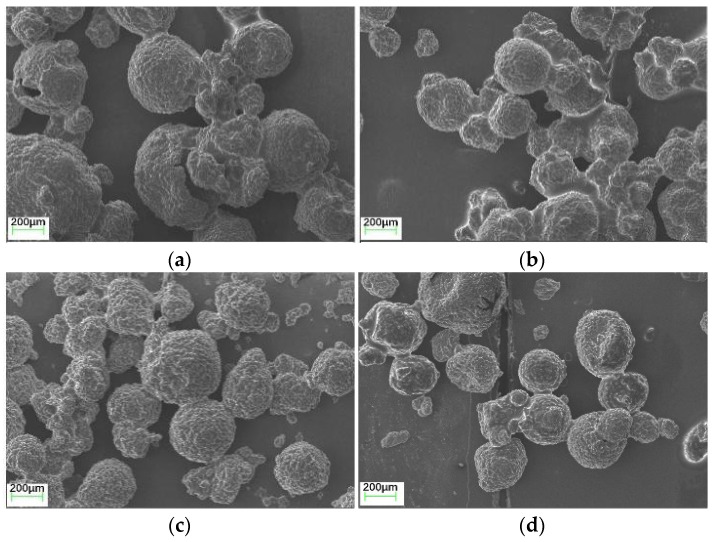
The morphology of microcapsules with different dosages of AGE. (**a**) MCs_20%_; (**b**) MCs_25%_; (**c**) MCs_30%_; (**d**) MCs_35%_.

Figure 5 and Figure 6 present the size distribution and SEM images of the microcapsules in four groups, namely, MCs_20%_, MCs_25%_, MCs_30%_, and MCs_35%_, to analyze the impact of diluent concentration on microcapsule preparation.

As shown in Figure 5, the specific surface area of the particle size distribution of the microcapsules demonstrated a trend of initially increasing and then decreasing with the increasing addition of AGE. The primary reason for this phenomenon is that an increase in AGE dosage leads to an improvement in the quality of the core material and an increase in molecular weight. According to dispersion theory, under constant stirring speed, a decrease in the specific surface area reflects a reduction in the dispersity of the oil particles. The oil phase particle size for AGE_30%_ and AGE_35%_ exhibited higher concentrations, resulting in a more focused distribution of microcapsule sizes compared to samples with lower AGE dosages. Furthermore, the decrease in specific surface area can slow down the rate of intermolecular chemical reactions. Due to the lower dispersity of the oil droplets, the adsorption efficiency of the emulsifier decreases, which is caused by the polymers produced during the polymerization reaction impeding the deposition of more polymers. Microcapsules with smaller particle sizes can disperse well within the matrix material, but may have an insufficient healing agent dosage, affecting the repair effectiveness. Conversely, larger particle sizes can store more healing agent, but may compromise the overall strength and durability of the material. Therefore, by adjusting the particle gradation, it is possible to achieve self-healing effects while simultaneously improving the overall strength of the material [72].

As shown in Figure 6, with the increase in AGE dosage, the aggregation of the microcapsules was reduced. This process is primarily driven by changes in the viscosity of the core material. Higher viscosity makes dispersion more challenging because the intermolecular forces exceed the internal friction forces caused by stirring in the liquid. Therefore, determining the optimal dosage of AGE is crucial to address the aggregation issue of microcapsules during synthesis. The figure also illustrates the rough and uneven surfaces of the microcapsules, which result from the deposition of urea–formaldehyde resin particles on the shell, forming textured surfaces. These textures enhance the adhesion between the microcapsules and the cement mortar [34,43]. Although the addition of AGE had a certain impact on the particle size and morphology of the microcapsules, studies have shown [12] that by adjusting the stirring rate to increase internal friction, the control over particle size range could be effectively managed.

#### 3.1.2. The Chemical Structure of Self-Healing Microcapsule Using FT-IR

**Figure 7:**
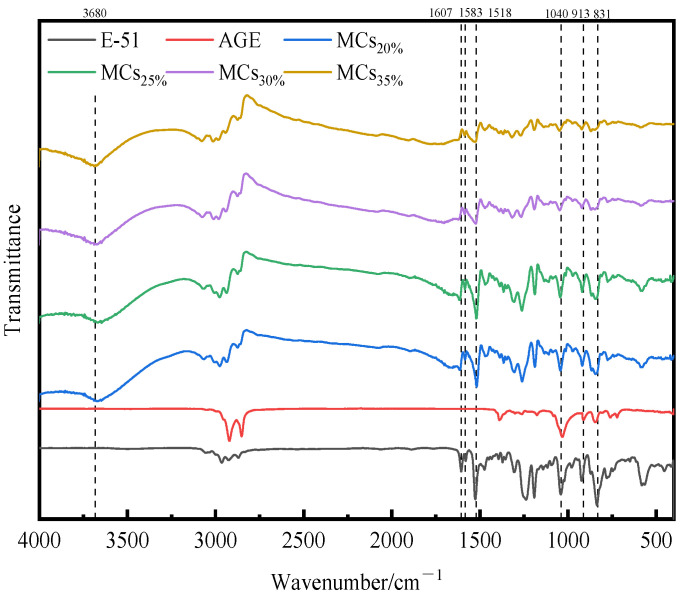
FT-IR spectra of microcapsules with different dosages of AGE.

Infrared spectroscopy analysis primarily identifies the components of the microcapsules to ensure the effective encapsulation of the core material by the wall material [73,74]. To evaluate the impact of the diluent addition on the chemical structure of the microcapsule core material, FT-IR spectroscopic analysis was conducted on four types of microcapsules made with E-51 and AGE, including MCs_20%_, MCs_25%_, MCs_30%_, and MCs_35%_, as shown in Figure 7. As a diluent, AGE primarily interacts with E-51 through physical interactions, reducing system viscosity and enhancing the flowability of E-51 [36]. The FT-IR spectra analysis for epoxy resin E-51, AGE, and microcapsules MCs_20%_, MCs_25%_, MCs_30%_, and MCs_35%_ reveals benzene ring vibrations at 1518 cm^−1^, 1583 cm^−1^, 1607 cm^−1^, and 831 cm^−1^, with the strongest absorption band at 831 cm^−1^, resulting from the asymmetric bending vibrations of the epoxy ring. Considering that the production conditions for the microcapsules were the same and that the mass of the core material is identical across different AGE dosages, the characteristic chemical formula of the epoxy resin in the core material decreases with increasing AGE addition. This is evidenced by the reduced quantity of the benzene ring absorption peaks at the aforementioned positions, confirming the effective encapsulation of the epoxy resin. Additionally, the characteristic peak values of the epoxy groups in AGE and E-51 are found at 913 cm^−1^ and the -C-O- stretching at 1040 cm^−1^, where the -C-O- is produced by the stretching vibrations of linear aliphatic ether structures and ether bonds within the epoxy resin. The absorption peaks at these two locations exhibit a similar variation pattern to those of the benzene ring, primarily because AGE contains only one epoxy group and -C-O-, while E-51 contains two. The decrease in their absorption peaks further validates the successful encapsulation of AGE and E-51. An absorption peak at 3680 cm^−1^ was observed, originating from the N–H bonds of the urea–formaldehyde resin wall material. The absorption peaks in the range of approximately 2900 cm^−1^ to 3000 cm^−1^ are primarily due to the stretching vibrations of aromatic and aliphatic C–H bonds.

#### 3.1.3. Thermal Stability of Self-Healing Microcapsule Using TGA

**Figure 8:**
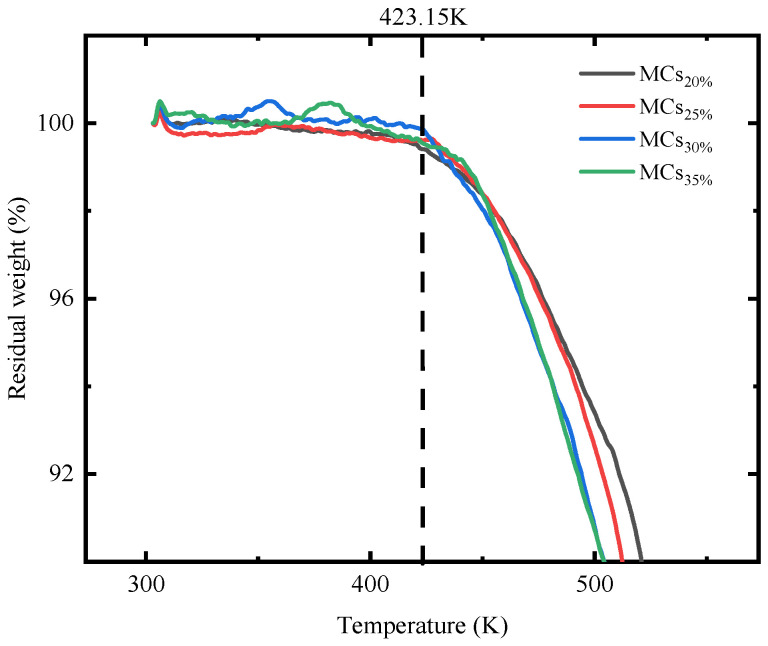
TGA results of microcapsules with different dosages of AGE.

The thermal stability of four types of microcapsules, including MCs_20%_, MCs_25%_, MCs_30%_, and MCs_35%_, was evaluated using a thermogravimetric analyzer (TGA). The TGA results, as shown in Figure 8, reveal a trend of decreasing residual mass with increasing temperature for all samples. Up to 423.15 K, the mass loss of the four samples was relatively small and similar in extent, primarily due to the evaporation of moisture within the microcapsules. However, as the temperature continued to rise, the trend in mass loss became more pronounced among microcapsules with different AGE dosages. The samples MCs_30%_ and MCs_35%_ exhibited a faster rate of mass loss compared to MCs_20%_ and MCs_25%_. This phenomenon suggests that microcapsules made from lower viscosity E-51 are more susceptible to mass loss at high temperatures, highlighting the relationship between the proportion of AGE added and the thermal stability of the microcapsules. Prior to 423.15 K, the microcapsules exhibit almost no mass loss, maintaining their structural integrity. This result is significant for the application of microcapsules during the hydration process of concrete, as the hydration heat of cement concrete ranges from 333.15 K to 343.15 K, with some reaching approximately 353.15 K [75,76,77]. The high thermal stability of the microcapsules indicates that they can withstand the high temperatures potentially encountered during the concrete production process, thereby maintaining their structural and functional integrity. This provides an effective material choice for self-healing technologies in concrete materials.

### 3.2. Influence of Different Diluent Concentrations of the Epoxy–Polythiol Self-Healing System

#### 3.2.1. Thermal Stability of Solidified Colloid in the Curing Reaction

**Figure 9:**
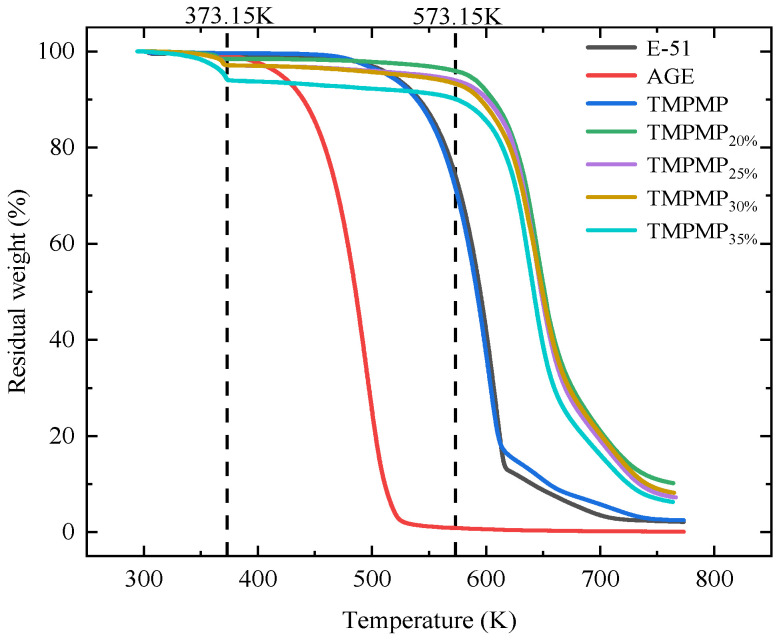
TGA results of the epoxy–polythiol self-healing system with different dosages of AGE.

Under the condition of a heating rate of 10 K/min, the thermal decomposition results of AGE, E-51, TMPMP_20%_, TMPMP_25%_, TMPMP_30%_, and TMPMP_35%_ are shown in Figure 9. The thermal stability of AGE is the poorest among the substances, while E-51 exhibits the best thermal stability, with degradation starting at 420.65 K and 517.15 K, respectively. The degradation temperature of TMPMP is close to that of E-51, occurring at 515.14 K.

Under stable heating conditions, the process of the mixture transitioning from a liquid to a solid state was observed, and the change in residual mass during this process was recorded using a thermogravimetric analyzer (TGA). This process can be divided into three distinct stages.

In the first stage (273.15 K–373.15 K), as the temperature rises initially, the mixture exhibits relatively stable characteristics with minimal mass change. This stage includes the evaporation of moisture from the mixture and some mass loss. The mixed solution can react at room temperature within 30 min, releasing a significant amount of heat that promotes the overall curing reaction. In the TGA tests, the gradual increase in temperature accelerates the curing reaction of the mixed solution, and the solution experiences mass loss near 373.15 K due to the combined effects of the test temperature and the heat released from the reaction.

In the second stage (373.15 K–573.15 K), after the curing reaction, the cured product experiences a slow mass loss as the temperature increases.

In the third stage (after 573.15 K), all samples exhibit a rapid decline in mass near 573.15 K as the temperature continues to rise.

The mass loss patterns in the second and third stages are similar to conventional TGA results [38,78]. The influence of different AGE dosages on the thermal stability of the epoxy resin–polythiol self-healing system is relatively minor, with the self-healing system remaining fundamentally stable before 573.15 K.

In this study, the temperature corresponding to a 5% mass loss of the mixture (T_d5%_) was used as a benchmark for evaluation [79,80]. Table 2 presents the starting degradation temperatures of each sample. The degradation temperatures of E-51 and TMPMP are similar, while the thermal degradation temperature of AGE is significantly lower than that of E-51 and TMPMP. Additionally, the thermal degradation temperatures of the mixtures TMPMP_20%_, TMPMP_25%_, TMPMP_30%_, and TMPMP_35%_ show a decreasing trend with increasing proportions of the diluent. The starting thermal degradation temperature for TMPMP_20%_ is 584.51 K, while that for TMPMP_30%_ is 530.17 K, indicating a relatively small decrease in temperature. In contrast, the starting thermal degradation temperature for TMPMP_35%_ is significantly lower, at only 370.95 K. The starting degradation temperatures of TMPMP_20%_ and TMPMP_25%_ are increased by 42.92% and 57.57%, respectively, compared to TMPMP_35%_.

#### 3.2.2. Effect of AGE Diluent Dosage on the Reaction Kinetics in the Curing System

**Figure 10:**
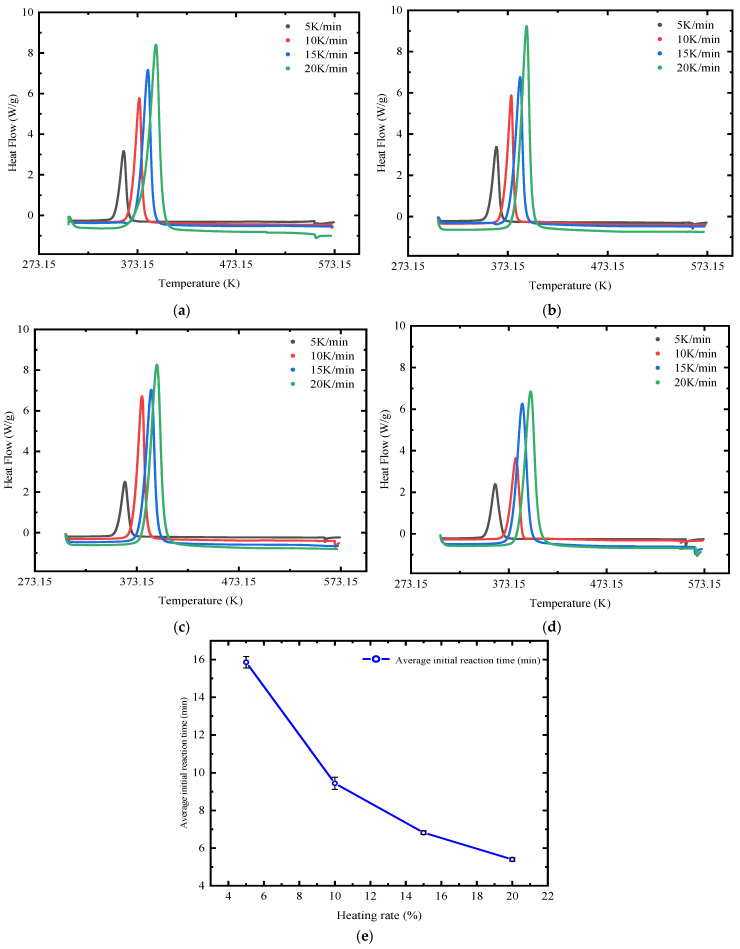
Dynamic curing DSC curves and average initial reaction time with various AGE dosages. (**a**) TMPMP_20%_; (**b**) TMPMP_25%_; (**c**) TMPMP_30%_; (**d**) TMPMP_35%_; (**e**) Average initial reaction time (min).

Differential scanning calorimetry (DSC) was used to obtain the thermal curves of TMPMP_20%_, TMPMP_25%_, TMPMP_30%_, and TMPMP_35%_ at different heating rates, as shown in Figure 10a–d. The results demonstrated the main exothermic peaks during the heating process, which became sharper as the heating rate increased.

The average initial reaction temperatures of the four samples at different heating rates are plotted in Figure 10e. It can be observed in the figure that the average initial reaction time of the curing process exhibited a decreasing trend with increasing heating rates, a phenomenon consistent with the typical behavior of thermosetting materials [19,81]. This indicates that in the self-healing system studied, the curing reaction predominantly occurs in one stage, and the reaction rate positively correlates with the increase in heating rate.

**Figure 11:**
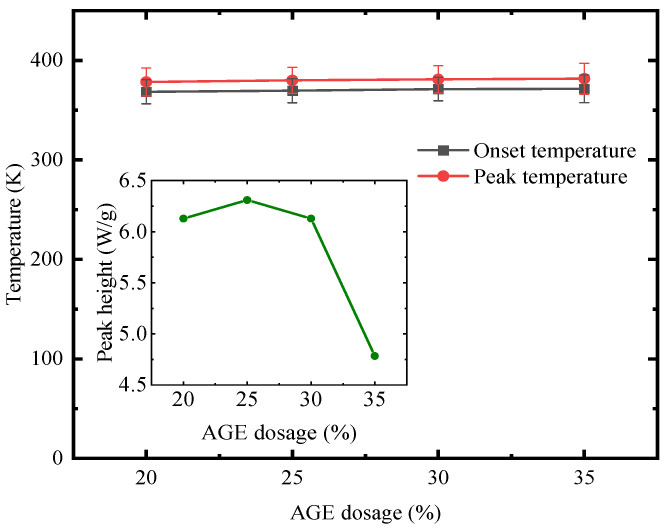
The effect of different AGE dosages on characteristic temperature and peak height.

The study found that varying the incorporation ratio of AGE causes changes in the onset and peak temperatures of the self-healing system. The averages of initial and peak temperatures of the four samples at different heating rates are shown in Figure 11, and the onset and peak temperatures of the curing reaction increased with the addition of AGE. This is due to AGE acting as an active diluent, which reduces the intermolecular forces between macromolecules in E-51, thereby affecting the curing reaction kinetics of the system. Further analysis indicated that the addition of AGE increases the volume of chemical groups in the mixture, requiring more energy consumption during the curing process. Within an AGE dosage range of 20–30%, the changes in peak temperature were relatively mild. However, at an AGE dosage of 35%, the peak temperature reached its minimum, indicating a more concentrated exothermic reaction. This suggests that a moderate addition of AGE can regulate the epoxy–polythiol curing reaction. However, when the AGE addition reaches 30–35%, the high concentration of epoxy groups to some extent disrupts the balance of the curing system, leading to a decreased exothermic rate compared to the 20–30% AGE ratio [82].

**Figure 12:**
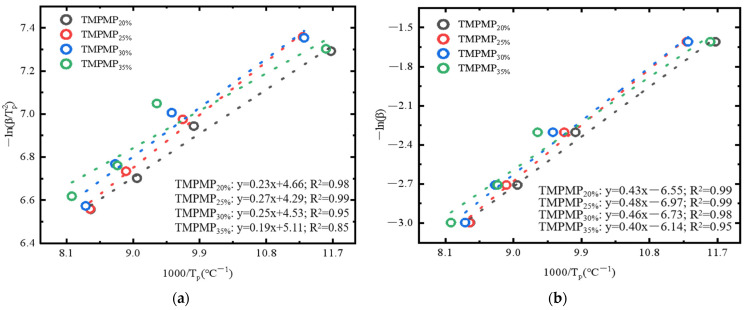
Linear fitting using the Kissinger and Crane equation. (**a**) $-\ln\left(\beta /T_{p}^{2}\right)$ vs. $1000/T_{p}$; (**b**) −lnβ vs. $1000/T_{p}$.

In this study, the Kissinger and Crane theoretical models were employed to investigate the effects of AGE dosage on the curing kinetics of the epoxy–polythiol self-healing system. The results indicated that the amount of AGE significantly impacts the thermokinetic properties. Figure 12 illustrates these findings. Figure 12a reveals the linear correlation between the change in heating rate and the thermokinetic parameters corresponding to the peak curing temperatures, showing a significant linear relationship between the −ln(*β*/($T_{p}^{2}$)) values and the 1000/$T_{p}$ values. Figure 12b displays a good linear relationship between −ln*β* and 1000/$T_{p}$. The correlation coefficient R^2^ values for both relationships were close to 1, validating the effectiveness of both models.

**Figure 13:**
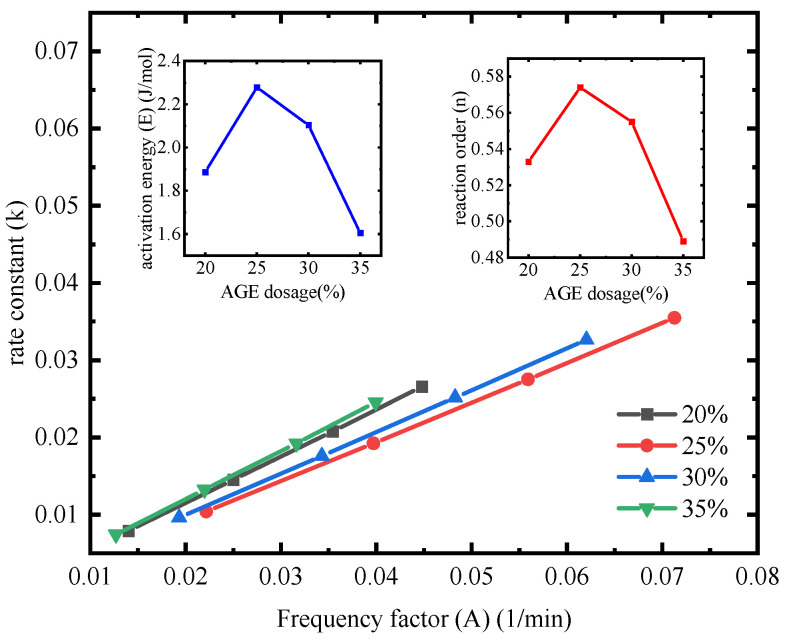
Kinetic parameters of the curing reaction with different dosages of AGE.

Figure 13 reflects the impact of varying AGE dosages on the kinetic parameters of the curing reaction. The results showed that TMPMP_20%_ and TMPMP_35%_ exhibit relatively lower activation energies, indicating higher reactive activity of the self-healing system at these incorporation ratios. However, the excessive addition of the diluent in TMPMP_35%_ increased the variation in cross-link density, leading to decreased reactivity in the curing reaction [82]. Therefore, to maintain the excellent performance of the self-healing system, it is recommended to control the AGE dosage between 20% and 30%.

The reaction order *n* characterized the complexity of the reaction mechanism. As the dosage of AGE increased, the change in reaction order was minimal, suggesting that AGE participates in the cross-linking reaction of the E-51 epoxy resin. The reaction orders under all AGE dosage were less than 1 and relatively close, indicating that the curing process involves multi-step complex chemical reactions, and the curing reaction characteristics of the self-healing system are essentially consistent.

The activation energy of the reaction showed a nonlinear variation with increasing AGE dosage, while the reaction order remained relatively stable. The frequency factor *A* was positively correlated with the heating rate, with the curing degree of TMPMP_20%_ being optimal. Meanwhile, the variation in the rate constant *k* of the curing reaction was minimal, indicating that the self-healing system can maintain stable reactive activity at room temperature or lower temperatures.

#### 3.2.3. The Effect of Different AGE Additions on the Young’s Modulus of the Self-Healing System

**Figure 14:**
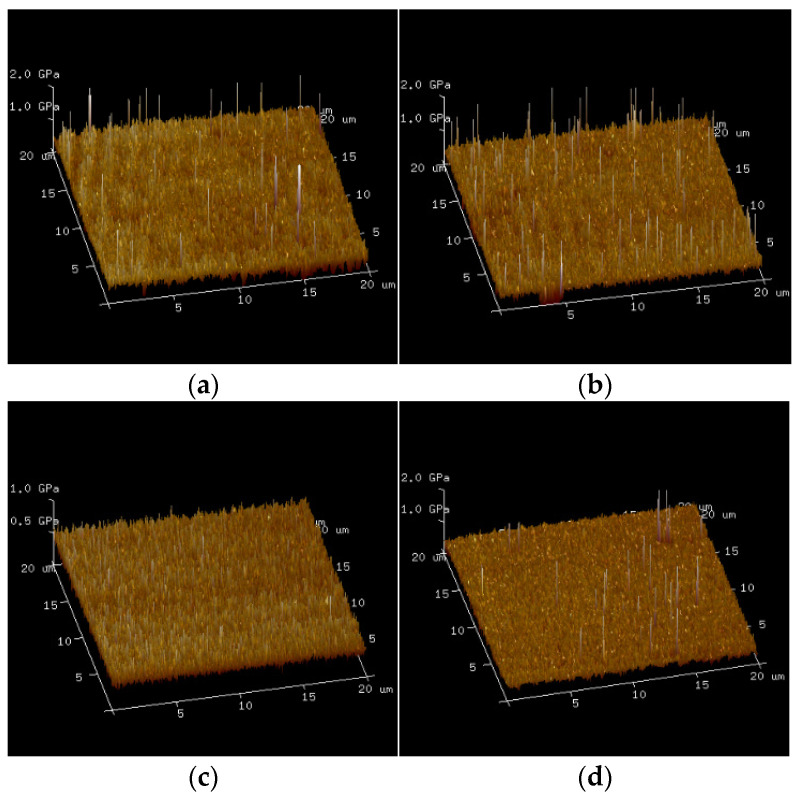
Three-dimensional diagram of equivalent Young’s modulus with various AGE dosages. (**a**)TMPMP_20%_; (**b**) TMPMP_25%_; (**c**) TMPMP_30%_; (**d**) TMPMP_35%_.

Figure 14 displays the 3D distribution of the equivalent Young’s modulus for TMPMP_20%_, TMPMP_25%_, TMPMP_30%_, and TMPMP_35%_ samples obtained using an atomic force microscope (AFM) at a scanning frequency of 0.40 Hz. It was observed that most of the Young’s modulus values are below 1 GPa, with the maximum values for the different samples being 56.806 GPa, 35.648 GPa, 2.681 GPa, and 47.808 GPa, respectively. The minimum values are 113.65 MPa, 91.39 MPa, 56.11 MPa, and 73.63 MPa, respectively. These data suggested that there were certain variations in material homogeneity within this self-healing system, with TMPMP_25%_ showing the most significant heterogeneity and TMPMP_30%_ exhibiting the best uniformity in the distribution of Young’s modulus. This heterogeneity could be due to several factors. These factors primarily originate from three areas: the quality of the raw materials used; the sample preparation methods, which might not have involved sufficient stirring during the process; and the differences in the microstructure of the self-healing system, thereby affecting the overall mechanical properties. Previous research has also found that epoxy resins with added diluents exhibit some degree of heterogeneity [82], which may be due to the diluent altering the cross-link density and network structure of the epoxy resin, negatively impacting the material’s mechanical properties [82,83,84], and thus affecting the uneven distribution of the Young’s modulus in the self-healing system.

**Figure 15:**
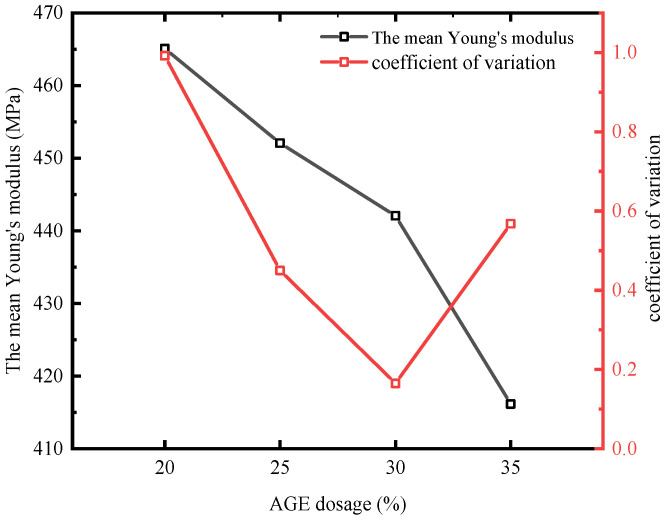
The mean Young’s modulus of TMPMP_20%_, TMPMP_25%_, TMPMP_30%_, and TMPMP_35%_.

Figure 15 shows that as the AGE dosage increases, the average Young’s modulus of the epoxy–polythiol self-healing system’s cured products in the test area exhibits a continuous decreasing trend. There was a significant negative correlation between the Young’s modulus and the increase in AGE dosage. This was particularly noted when the AGE dosage increased from 30% to 35%, and the reduction in Young’s modulus was most pronounced at 5.88%. This reduction may be attributed to the role of AGE in the self-healing system, as the amount of AGE increases, the cross-link density and network structure continually change, subsequently affecting the material’s rigidity [84,85]. The coefficient of variation (CV) is defined as the ratio of the standard deviation to the mean of a dataset and is used to measure the degree of data dispersion. In this study, when the AGE dosage is 20%, the CV reached its maximum value, approaching 1, indicating a high degree of variability in the Young’s modulus. When the AGE dosage is 30%, the CV decreased to a minimum value of 0.165, reflecting reduced dispersion. This trend is consistent with the observations presented in Figure 14. In cement concrete structures, high stiffness and load-bearing capacity are crucial performance indicators. Thus, although the addition of a diluent can enhance the material’s flowability and improve its crack healing effect [47], excessive addition may compromise the mechanical properties [82,83,84]. Therefore, studying the specific impacts of different types and dosages of diluent on the mechanical properties of epoxy–polythiol cured products to find an optimized ratio that achieves the best balance between self-healing ability and mechanical properties is of significant importance for the development of future high-performance self-healing materials.

## 4. Discussion

This study aims to find the optimal ratio between epoxy resin and AGE using viscosity testing, microcapsule characterization, thermodynamics, and mechanical performance analysis, achieving the best balance between self-healing capability and mechanical properties, thereby providing a reference for the development of high-performance self-healing materials. In this research, Section 2.2 explores the diluting effect of AGE on E-51 using viscosity tests, preliminarily determining the range of optimal incorporation. Section 3.1 utilizes characterization techniques such as SEM, LPA, FT-IR, and TGA to provide data support [86,87], discussing the influence of AGE incorporation on the basic properties of E-51 microcapsules. Section 3.2 analyzes the key roles and impact characteristics of AGE in solutions mixed with different qualities of AGE, E-51, and TMPMP during and after the curing reaction, exploring the significant effects of AGE incorporation on the thermodynamic properties of the epoxy–thiol self-healing system based on Equations (1)–(5). Additionally, analysis using Equations (6)–(8) indicates that the incorporation of AGE also affects the rigidity of the self-healing system.

The principle of mechanically triggered microcapsule self-healing technology is that when cracks form in concrete, they encounter microcapsules. The stress concentration at the crack tips triggers the rupture of the microcapsules, causing the core material to flow out and, under capillary action, move toward the cracks to fill and repair them. However, challenges remain in practical applications. The healing agent E-51 and the curing agent TMPMP are not always mixed in a 1:1 mass ratio, and there may be uneven self-healing effects. Moreover, the residual capsules from broken microcapsules may affect the overall performance of the cured products and structures. This study primarily investigates the role of the environmentally friendly diluent AGE in the entire self-healing system of epoxy resin and thiol, covering aspects from core material flow characteristics to the properties of the microcapsules themselves, as well as the molecular dynamics during the curing reaction of the AGE, E-51, and TMPMP mixed solution and the mechanical properties of the cured products, without resolving all issues encountered in practical engineering. In light of these challenges, to further determine the optimal ratio of epoxy resin and diluent for achieving the best healing rate, it is recommended to design experiments incorporating epoxy resin microcapsules with varying AGE dosages into cement-based materials. This should be combined with mechanical testing, durability testing, and non-destructive testing using extensive repeat experiments to identify the optimal material ratios. This will become a focal point of future research efforts.

## 5. Conclusions

Based on the viscosity test results, the optimal dosage range of AGE incorporated in epoxy resin is between 20% and 35%. Therefore, AGE dosages of 20%, 25%, 30%, and 35% were selected herein to explore the effects of the diluent AGE on the microencapsulation and the performance of the epoxy–polythiol self-healing system. Major findings are concluded as follows.

(1) The incorporation of AGE has a noticeable impact on the particle size distribution, chemical bonding, and thermal stability of the microcapsules. With an increase in AGE dosage, the particle size distribution of the microcapsules becomes more concentrated, and the particles become more dispersed. Infrared spectroscopy analysis confirmed that E-51 and AGE were successfully encapsulated. Regarding thermal stability, MCs_20%_, MCs_25%_, MCs_30%_, and MCs_35%_ maintain their structural integrity before 423.15 K. This result is significant for the role of microcapsules in resisting the heat of hydration during the concrete hydration process (≈337.15 K).
(2) The incorporation of AGE significantly affects the thermal stability of the epoxy–polythiol self-healing system. As the temperature increases, the mixture transitions from a liquid to a solid state at 373.15 K, releasing heat that accelerates the thermal degradation of the material. The initial degradation temperature of the mixture shows a decreasing trend with increasing AGE dosage. The initial degradation temperature of TMPMP_35%_ is 370.952 K, while that of TMPMP_20%_ is 584.51 K, representing an increase of 57.57% compared to TMPMP_35%_. Additionally, the initial and peak temperatures of the curing reaction increase with the amount of AGE incorporated.
(3) The incorporation of AGE significantly affects the thermokinetic properties. The study found a significant linear relationship between the heating rate and the thermokinetic parameters corresponding to the peak curing temperatures. The activation energy (*E*) exhibited a trend of initially increasing and then decreasing with the addition of AGE, with TMPMP_25%_ having the highest activation energy. Additionally, the frequency factor (*A*) is positively correlated with the heating rate, indicating that the self-healing system can maintain stable reactive activity at room temperature or lower temperatures, although the changes in the rate constant (*k*) are small.
(4) The addition of AGE has a certain impact on the rigidity of the self-healing system. The average Young’s modulus shows a significant negative correlation with the amount of diluent added, with the most significant decrease (by 5.88%) occurring when the AGE dosage increases from 30% to 35%. This is because the addition of diluent affects the cross-link density of the self-healing system, which has a negative effect on its mechanical properties.

Therefore, an appropriate amount of AGE can effectively optimize the performance of the self-healing system, making it more suitable for use in demanding engineering applications. Future research can further explore how to balance these physical and chemical properties to achieve application under a broader range of environmental conditions.

## Figures and Tables

**Table 1:** Microcapsule formulation with a diluent dosage of 30%.

Materials	Component
Sodium dodecyl benzene sulfonate 0.75 wt% (AR)	150 mL
Urea (AR, 99%)	10 g
Ammonium chloride (AR, 99.5)	1 g
Resorcinol (AR, 99%)	1 g
Formaldehyde (AR, 37% in H_2_O)	27.1 g
Epoxy resin (E-51)	50 g
C12-14 alkyl glycidyl ether (AR, epoxy value: 0.3–0.34)	15 g
n-Octanol (AR, 99%)	Quantum satis
Citric acid 10 wt%	Quantum satis
Ethanol (AR, 75% in H_2_O)	Quantum satis

**Table 2:** Starting thermal degradation temperatures of samples.

Sample	T_d5%_ (K)
AGE	420.65
E-51	517.15
TMPMP	515.14
TMPMP_20%_	584.51
TMPMP_25%_	548.23
TMPMP_30%_	530.17
TMPMP_35%_	370.95

## Data Availability

Data are contained within the article.
